# High-Efficiency Automatic Recharging Mechanism for Cleaning Robot Using Multi-Sensor

**DOI:** 10.3390/s18113911

**Published:** 2018-11-13

**Authors:** Ching-Lung Chang, Chuan-Yu Chang, Zhi-Yuan Tang, Shuo-Tsung Chen

**Affiliations:** 1 Department of Computer Science and Information Engineering, National Yunlin University of Science and Technology, Yunlin 64002, Taiwan; chang@yuntech.edu.tw (C.-L.C.); chuanyu@yuntech.edu.tw (C.-Y.C.); M10517021@yuntech.edu.tw (Z.-Y.T.); 2 Intelligent Recognition Industry Service Research Center (IRIS Research Center), National Yunlin University of Science and Technology, Yunlin 64002, Taiwan; 3 College of Future, Bachelor Program in Interdisciplinary Studies, National Yunlin University of Science and Technology, Yunlin 64002, Taiwan

**Keywords:** cleaning robot, automatically returns to charging, infrared spots, six-axis acceleration, ultrasound

## Abstract

Cleaning robot has the highest penetration rate among the service robots. This paper proposes a high-efficiency mechanism for an intelligent cleaning robot automatically returns to charging in a short time when the power is insufficient. The proposed mechanism initially combines the robot’s own motor encoder with neural network linear regression to calculate the moving distance and rotation angle for the location estimation of the robot itself. At the same time, a self-rotating camera is applied to scan the number of infrared spots on the docking station to find the location of the docking station so that the cleaning robot returns to charging properly in two stages, existing infrared range and extended infrared range. In addition, six-axis acceleration and ultrasound are both applied to deal with the angle error that is caused by collision. Experimental results show that the proposed recharging mechanism significantly improves the efficiency of recharging.

## 1. Introduction

In recent years, mobile robots are being widely used in industrial automation, home automation, hospitals, entertainment, space exploration, military, etc [1,2,3,4,5,6,7,8,9,10,11,12,13]. Due to the decreased size and the low cost of mobile robots, more and more mobile robots are now working around us and they will help us a lot in our daily lives, such as cooking, cleaning, house plant watering, pet feeding, and taking care of children [1,2,3,4,5]. In order to complete these tasks continuously and efficiently, many auto-recharging methods have been proposed.

Currently efficient auto-recharging methods can be generally classified into three types. (1) In simultaneous localization and mapping (SLAM), the robot starts from an unknown environment and acts in this environment, and continuously repeats the information of the environment to achieve its own positioning and status. Then, build an environmental map one by one based on the surrounding environment information constructed by its own location [14,15,16,17,18,19]. Lidar SLAM has high measurement accuracy but is expensive. Camera SLAM is cheaper but the disadvantage is that if the image is large, the calculation is complicated. (2) In wireless positioning and recharging, the strength of the received wireless signal is used to estimate the position of the docking station by using wireless signals to sense the position of the docking station, such as Bluetooth, WiFi or ZigBee wireless communication [20,21,22,23]. Depending on the wireless device, the requirements of the corresponding environment are different. Therefore, this method is susceptible to environmental factors. Especially in the same frequency band in the same area, the signal strength will be seriously affected. (3) In combination with various types of sensor for positioning and recharging, the recharging path is determined by sensing the position/orientation of the cradle by sensors, such as ultrasound, camera, infrared, etc [24,25,26,27,28,29]. The advantage of this method is that it can integrate the information collected by multiple sensors, and the obtained positioning accuracy is more accurate than the information collected by a single sensor. In addition, the cost of this method is much lower than using the laser radar SLAM.

Based on the review and discussion above, this paper uses multiple sensors to propose a high-efficiency automatic recharging mechanism for cleaning robot. Cleaning robots are the most widely used in family. For cleaning robots, there are general types and intelligent ones. The general-type cleaning robot has basic functions, including cleaning and recharging without positioning and path planning. Intelligent cleaning robots are equipped with sensors, such as ultrasound, camera, infrared, etc. These sensors are applied to complete the robot’s positioning and path planning. With various sensors, ultrasound, camera, infrared, six-axis acceleration, we design a high-efficiency mechanism of positioning and path planning for an intelligent cleaning robot automatically returns to charging in a short time when the power is insufficient. The proposed mechanism initially combines robot’s own motor encoder with neural network linear regression to calculate the moving distance and rotation angle for the location estimation of the robot itself. In order to adjust the travel direction of the robot until it enters the exiting infrared signal range, a self-rotating camera is applied to scan the number of infrared spots on the docking station so that the cleaning robot moves toward the infrared spots until the infrared spot changes from one to two, indicating that it enters the exiting infrared signal range (60 degree range) since there is only one infrared spot captured in the extended infrared range of 90 degrees. According to the robot’s speed, both six-axis acceleration and ultrasound are hierarchically applied to solve the problem that is caused by collision. Six-axis sensor is enabled to compensate the angle error that is caused by collision when the travel speed is slow while ultrasonic is enabled to avoid obstacles when the travel speed is fast.

The rest of this paper is organized as follows. Section 2 lists the specification of the test cleaning robot and hardware setting. Section 3 presents the proposed recharging mechanism. Section 4 shows experimental results. Section 5 concludes our work.

## 2. Introduction to the Testing Cleaning Robot and Hardware Setting

### 2.1. Introduction to the Testing Cleaning Robot

**Figure 1:**
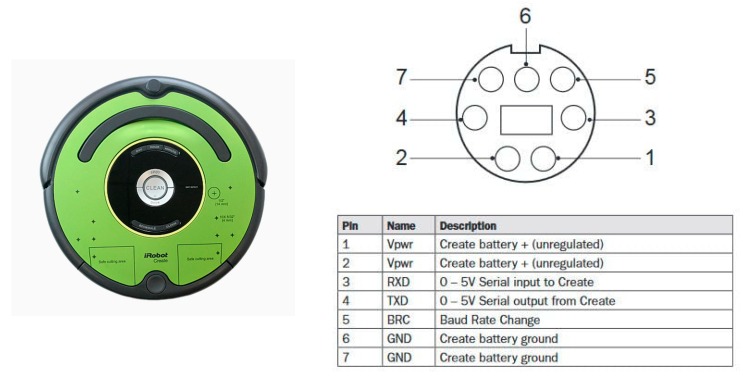
IRobot Create 2.

**Figure 2:**
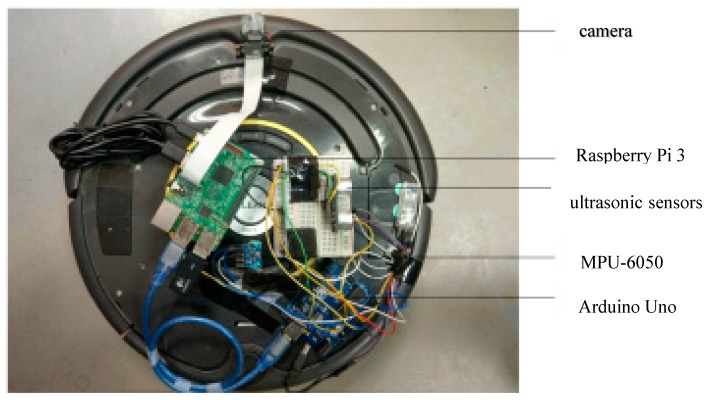
Cleaning robot hardware architecture.

The testing robot in this paper is a teaching robot produced by American IRobot company, as shown in Figure 1. User communicates with the robot through RS-232 communication interface. Based on this robot, we combine Arduino platform, Raspberry Pi 3, cameras, and ultrasonic sensors to design an automatic return charging mechanism. Figure 2 shows the overall structure of the robot. The hardware setting and the proposed mechanism are introduced in the following subsections.

RS-232 is a serial-type data transmission. It is a bit-by-bit character transfer method. Most of them use the RX and TX pins to communicate with each other. The robot uses the RX to receive the instructions from the Arduino and the TX transfers the state of the robot to the Arduino. The robot has three modes: (1) Passive mode, enable the robot, but cannot modify the parameters of the internal sensor. (2) Safe mode, enable the robot, and modify the parameters of the internal sensor, but some sensors will be restricted. (3) Full mode, has the second function above and no restrictions of sensor modification. 

This paper adopts mode (3). Based on the official specifications [5], its control instruction is Opcode (Open Interface control instruction), including Start, Stop, Drive, Drive Direct, which represent start, stop, forward/backward, and rotation, respectively. These commands are used to control robot. We set the speed value of the wheel, so that the robot can travel at this speed and turn left/right. For example, if the speed value is 50, then the robot advances at a speed of 50 mm/s. 

In the sweeping function, the clean robot has three functions: random-path cleaning, spiral-path cleaning, and square-path cleaning. In the part that automatically returns to the charging, the signal that is emitted by the infrared sensor of the docking station is used, and during the traveling, the infrared signal is continuously scanned to guide the cleaning robot to return to the docking station for charging.

### 2.2. Arduino for Calculating Travel Distance and Direction Angle

**Figure 3:**
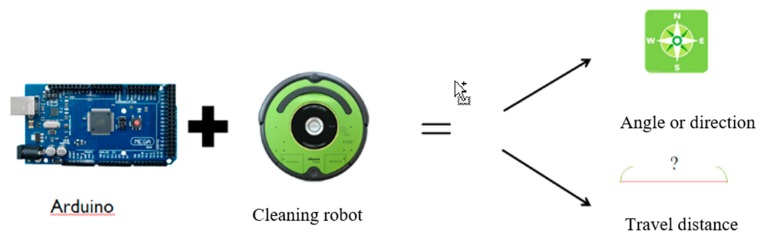
Arduino is used as the platform for calculating travel distance and direction angle.

The general mechanism of the cleaning robot cannot know its position when performing work tasks. When returning to the docking station for charging, it can only rely on the only infrared scanning function, but even if the infrared scanning function is used, the cleaning robot is not in the docking station. In the range of the infrared signal sent, it takes a long time to recharge. In order to solve this problem, the embedded platform Arduino is used as the platform for robot control. Through this platform, the travel distance and direction angle are calculated, as shown in Figure 3.

### 2.3. Infrared LED and Camera

**Figure 4:**
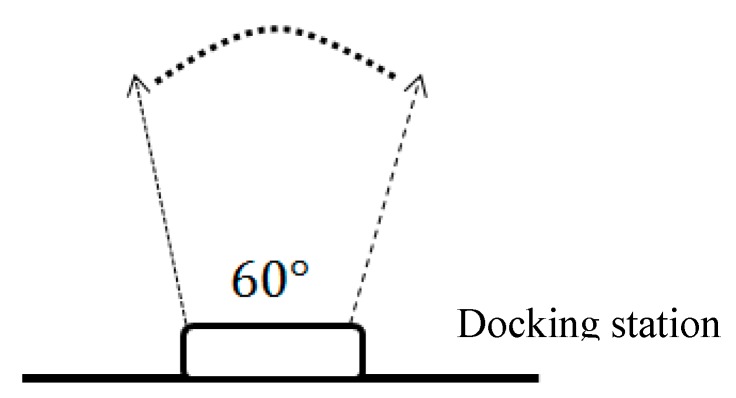
Exiting range of infrared light.

The automatic back to charging method of the clean robot is to continuously scan the infrared sensor signal on the charging stand by using the infrared sensor on the clean robot. Figure 4 shows the emission range of the infrared light. In this range, the clean robot can return to charging once it detects the infrared signal. However, outside the range, the robot itself does not know that the docking station is beside and thus walks randomly or even away from the docking station.

**Figure 5:**
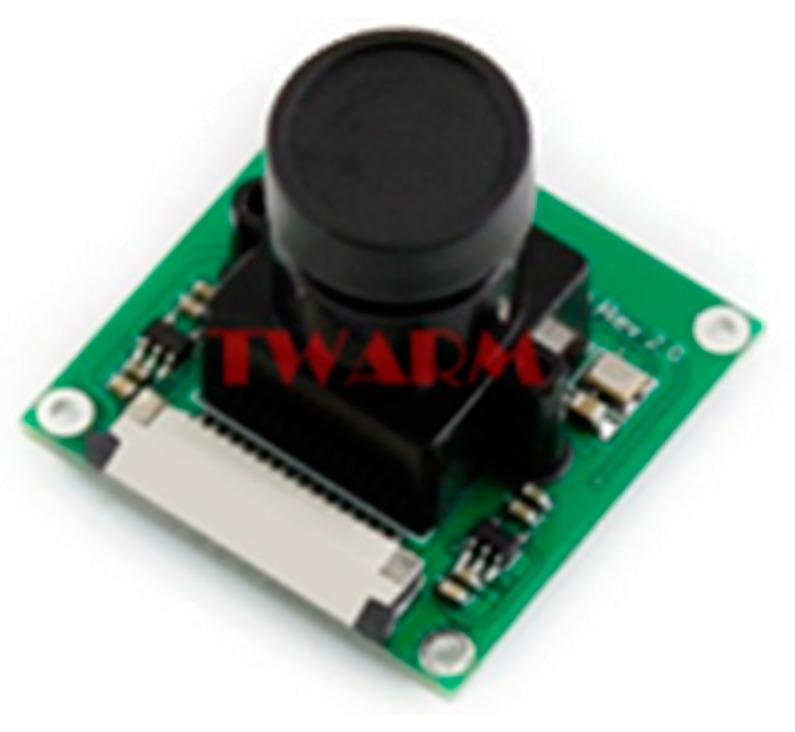
Camera.

**Figure 6:**
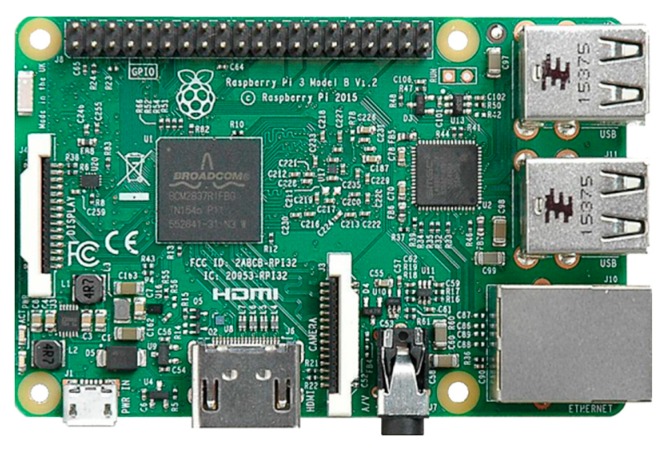
Raspberry Pi 3.

In order to improve the above situation, we added three infrared LEDs to the docking station to increase the exiting infrared signal range. Since the infrared sensor cannot detect the added infrared LED, it is necessary to use the camera and process the image captured with the Raspberry Pi 3, as shown in Figure 5 and Figure 6.

**Figure 7:**
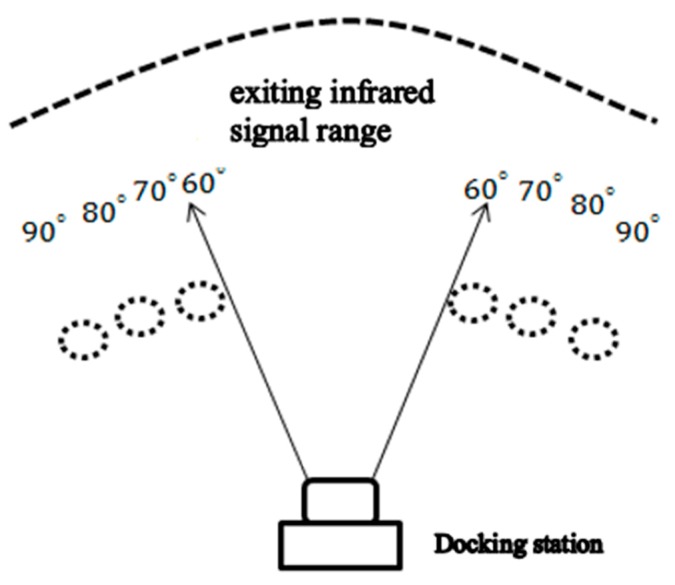
Infrared signal range with and without adding three infrared LEDs.

In order to confirm the range of the camera after adding the camera, as shown in Figure 7, the black dotted circle is the position where the cleaning robot is placed, and the upper figure is the angle corresponding to the docking station. We first placed the robot from the middle of the 70-degree position, and watched whether the camera had infrared light. After testing, it was found that the range of shooting was up to 90 degrees.

If you want to increase the range to more than 100 degrees, then you need to add more LEDs, but in fact the 90-degree range is enough for the robot to return to the docking station correctly, as shown in Table 1.

### 2.4. Six-Axis Sensors

**Figure 8:**
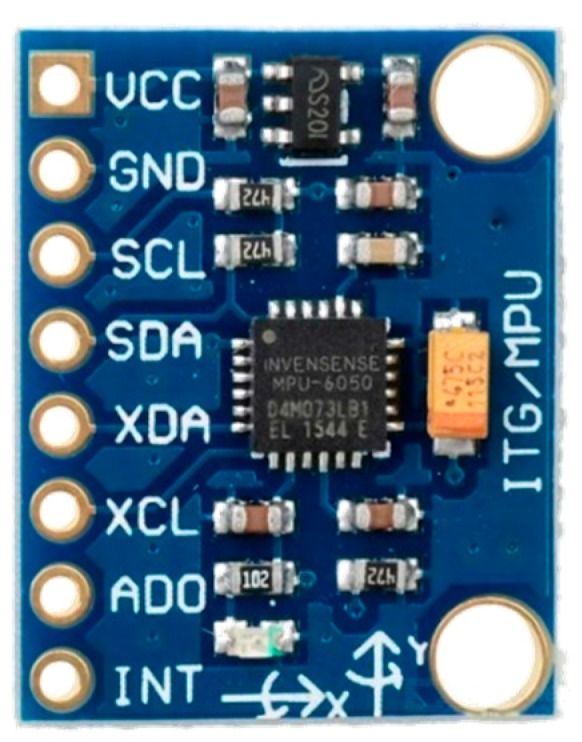
MPU-6050 six-axis sensor.

The MPU-6050 six-axis sensor, as shown in Figure 8, is composed of an accelerometer and a gyroscope that calculate the current direction or angle of the robot. Specifications are shown in Table 2.

## 3. Proposed Mechanism

This section mainly combines neural network-linear regression with the proposed sensing techniques, including self-rotating camera, six-axis sensors, and ultrasonic sensors to propose a high-efficiency recharging mechanism.

### 3.1. Neural Network-Linear Regression

We review neural network in case of linear regression for later use before introducing the proposed mechanism for a clean robot automatically returns to charging. A neuron with label $y_{j}$ receives an input $x_{i}$ from predecessor neurons by a connection assigned a weight $w_{ji}$.

(1) $$y_{j}=\sum_{i}w_{ji}x_{i}=W_{j}^{T}X$$

where $W_{j}={\left[\begin{array}{ccc} \cdot \cdot \cdot & w_{ji} & \cdot \cdot \cdot \end{array}\right]}^{T}$ and $X={\left[\begin{array}{ccc} \cdot \cdot \cdot & x_{i} & \cdot \cdot \cdot \end{array}\right]}^{T}$. The error $e_{j}$ between real output $d_{j}$ and $y_{j}$ is then rewritten as

(2) $$e_{j}=d_{j}-y_{j}=d_{j}-W_{j}^{T}X$$

or equivalently

(3) $$\frac{1}{2}e_{j}^{2}=\frac{1}{2}{\left(d_{j}-W_{j}^{T}X\right)}^{2}$$

The total error is

(4) $$\frac{1}{2}e^{2}=\frac{1}{2}\sum_{j}e_{j}^{2}=\frac{1}{2}\sum_{j}{\left(d_{j}-W_{j}^{T}X\right)}^{2}$$

which implies

(5) $$\frac{1}{2}\frac{\partial e^{2}}{\partial W_{j}}=-\sum_{j}(d_{j}-W_{j}^{T}X)X=-eX$$

By introducing a parameter η called learning rate, the new weighting $W_{j}^{new}$ is obtained by

(6) $$W_{j}^{new}=W_{j}-(-\eta eX)=W_{j}+\eta eX$$

**Figure 9:**
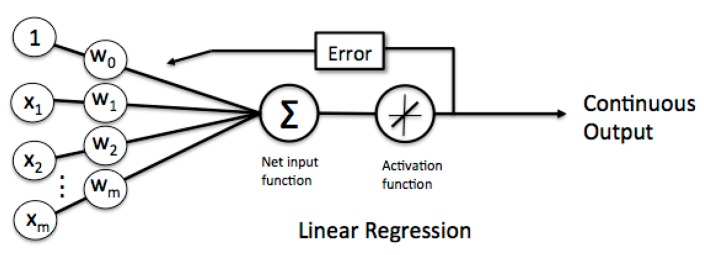
Machine learning-linear regression.

As shown in Figure 9, this expression means that each input will correspond to a different weight, and the output is multiplied by the weight to calculate the input of the next layer, and the weight is continuously updated until convergence to obtain the continuous output finally.

#### 3.1.1. Travel Distance Estimation

The distance *D* traveled by the cleaning robot is generally defined by

(7) $$D=v(d_{t}/100)$$

where *v* is the speed value of the wheel; $d_{t}$ is the duration of the walking (in milliseconds); and, $d_{t}/100$ denotes time calculation unit of 100 milliseconds. Replacing $d_{t}/100$ by *t*, equation (7) can be rewritten as

(8) D(t)=vt

**Figure 10:**
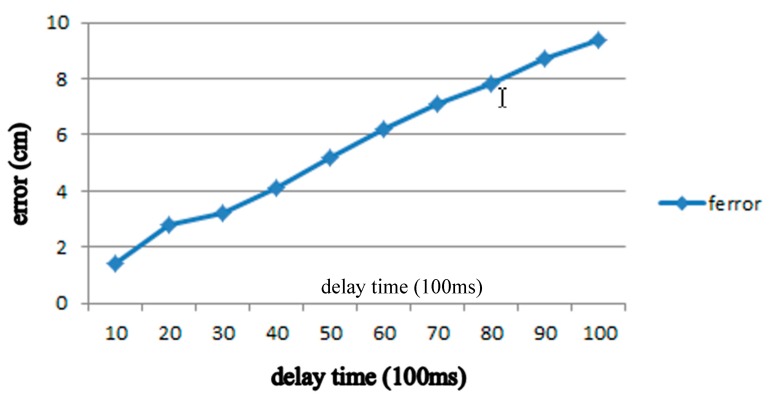
$f_{error}$ curve.

In other words, 1 s is equal to 1000 ms/100 ms = 10 which means *t* = 10. If the travel time is 2 s then D(2)=v(2×10). In order to verify the efficiency of formula in (2), we perform a series of experiments and the results are shown in Table 3. By the relation, distance error = actual travel distance − estimated travel distance, these results indicate that the longer the time, the greater the error, as shown in Figure 10. 

In order to reduce the distance error in Table 1, we apply neural network-linear regression in section A to find the curve $f_{error}$ in Figure 6 sketched by the distance error by

(9) $$f_{error}=\sum_{i}w_{i}x_{i}=W^{T}X$$

(10) $$W^{new}=W-(-\eta eX)=W+\eta eX$$

where $W={\left[\begin{array}{ccc} \cdot \cdot \cdot & w_{i} & \cdot \cdot \cdot \end{array}\right]}^{T}$, $X={\left[\begin{array}{ccc} \cdot \cdot \cdot & x_{i} & \cdot \cdot \cdot \end{array}\right]}^{T}$, and η=0.0001.

When the number of iterations reaches 500 times, the error has become saturated. Therefore, we train 500 times and compare the estimated travel distance with the actual travel distance. The results are shown in Table 4. The estimated distance between the traveled distance and the actual travel distance can be improved a lot. However, when the walking time reaches 30 to 40 s, the actual travel distance is gradually smaller than the estimated travel distance, but, when considering that the robot used in the home is not Continuous straight line travels for 30 s, and the method of this method will recalculate the distance as long as the robot collides or detects obstacles, so the part after 30 s is not considered.

#### 3.1.2. Rotation Angle Estimation

To get the location of the robot in addition to the estimated travel distance, we need to know the rotation angle of robot’s body itself by the formula in Equation (11).

(11) $$\theta =(\frac{delay}{100})(\frac{\left|E_{R}-E_{L}\right|}{L})$$

where *θ* is the rotation angle of the robot; $E_{R}$ is the speed value of the right wheel; $E_{L}$ is the speed value of the left wheel; and, L is the distance between the left wheel and the right wheel. The time part of formula (11) can also be replaced by t according to the method of the previous section, and can be obtained formula (12).

(12) $$\theta (t)=(\frac{\left|E_{R}-E_{L}\right|}{L})$$

**Figure 11:**
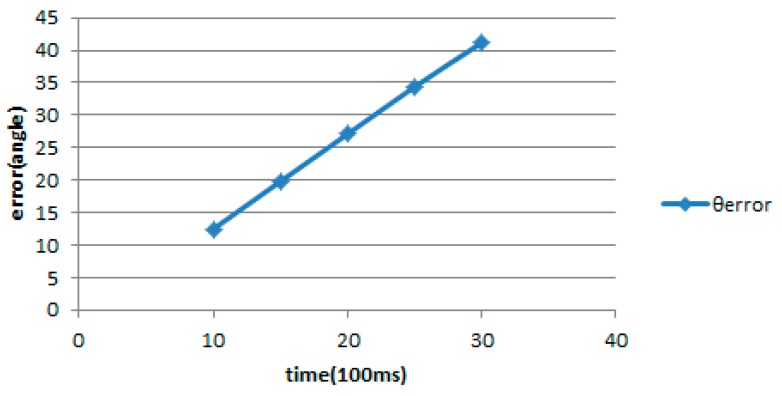
$\theta_{error}$ Graph.

In order to verify the efficiency of the formula (12), we have a series of tests and the results are shown in Table 5. From the results, the longer the duration of rotation, the angle error becomes ever larger. According to the estimated rotation angle, the actual rotation angle is smaller than the estimated rotation angle, so that we have the relation *actual rotation angle = estimated rotation angle − angle error*. In order to reduce the angle error in Table 5, we apply neural network-linear regression in section A to find the curve $\theta_{error}$ in Figure 11 sketched by the angle error as the following two formulas.

(13) $$\theta_{error}=\sum_{i}w_{i}x_{i}=W^{T}X$$

(14) $$W^{new}=W-(-\eta eX)=W+\eta eX$$

where $W={\left[\begin{array}{ccc} \cdot \cdot \cdot & w_{i} & \cdot \cdot \cdot \end{array}\right]}^{T}$, $X={\left[\begin{array}{ccc} \cdot \cdot \cdot & x_{i} & \cdot \cdot \cdot \end{array}\right]}^{T}$.

When the number of trainings is 300 to 500 times, the resulting angular error is already close to zero. Therefore, we train 300 times and compare the estimated rotation angle with the actual rotation angle. The results are shown in Table 6. The estimated distance between the traveled distance and the actual travel distance can be improved a lot.

### 3.2. Cleaning Robot Position Estimation

**Figure 12:**
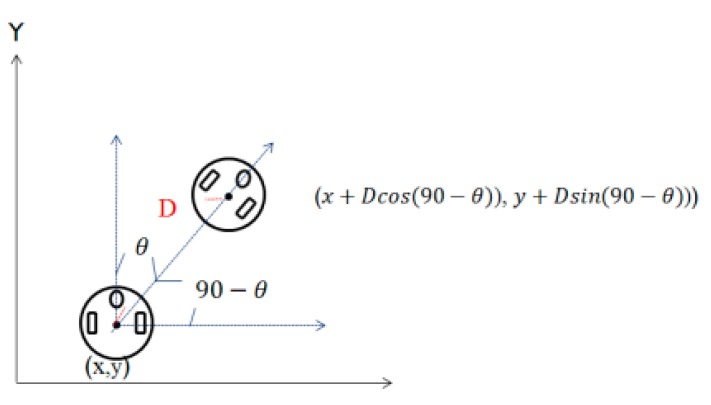
Robot position estimation.

After we estimate the distance and rotation angle of the robot by compensation, we can estimate the position of the robot. Based on the Cartesian coordinate system, the location of the docking station is set to (0, 0) and then the position of the robot is calculated by the following formula.

(15) $$\{\begin{array}{c} x=x+D\cos(90-\theta )\\ y=y+D\sin(90-\theta ) \end{array},\;(0\leq \theta \leq 2\pi )$$

where *D* corresponds to the travel distance and 90−θ is the rotation angle between the robot and the x-axis, as shown in Figure 12. 

**Figure 13:**
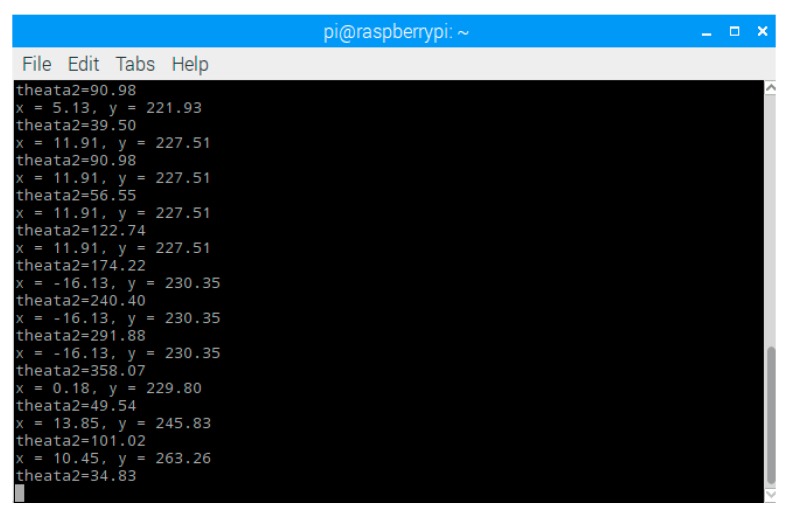
Remote observation interface.

The compensation of travel distance and rotation angle are calculated in Arduino to obtain robot’s Cartesian coordinate. In addition, we use USB to connect Arduino and Rasberry pi, so that we can observe robot’s Cartesian coordinate on end device by Rasberry pi, as shown in Figure 13.

### 3.3. Self-Rotating Camera for Searching Docking Station

In order to adjust the robot until it enters the exiting infrared signal range, we propose a searching method for the self-rotating camera combined with the angle and distance estimation proposed in the previous section. This search method is described, as follows. When the infrared spot is captured via the HoughCircles function [30], it is judged whether the docking station is currently on the left or the right side of the robot according to the current estimated angle and distance, and proceeds in the direction of the judgment. This method will be used repeatedly until the infrared spot changes from one to two, indicating that it enters the infrared signal range (60° range), because in the 90° range, only one infrared spot is captured. Moreover, we can compare the actual distance between the robot and the docking station from Table 7, thereby eliminating the distance error.

### 3.4. Collision Angle Error Compensation by Six-Axis Sensors

**Figure 14:**
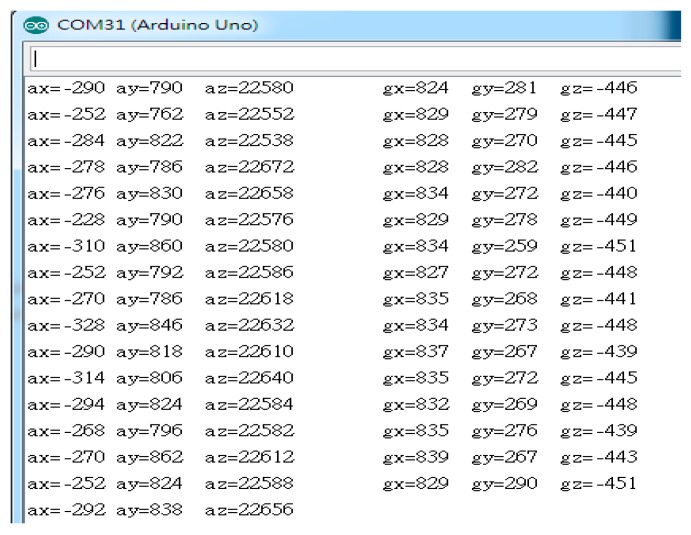
Six-axis sensor raw value.

During the execution of the mission, the clean robot may collide with obstacles. When it hits an obstacle, the robot may have a collision displacement. This collision displacement will cause the robot’s angle to change. In order to correct the collision offset, this paper uses six-axis sensors (MPU-6050) to compensate for collision error angles. As shown in Table 8, there are about 10° of error between estimated angle and actual angle due to the collision. In order to reduce the error, we adopt six-axis sensor with two parts: accelerometer and Gyroscope. The values read by the accelerometer and Gyroscope in the six-axis sensor are shown in Figure 14.

#### 3.4.1. The Part of the Accelerometer

Since the clean robot is traveling horizontally, we calculate the angle $\phi_{acce}$ between the *Z*-axis and the *X*-axis of the accelerometer by using the values read by the accelerometer in the six-axis sensor, *az* and *ax*, respectively.

(16) ϕacce=tan−1(aczacx)×180π

where *acz = az/accesen*, *acx = ax/accesen*, and *accesen* is the sensitivity of the accelerometer with unit LSB/g.

#### 3.4.2. The Part of the Gyroscope

**Figure 15:**
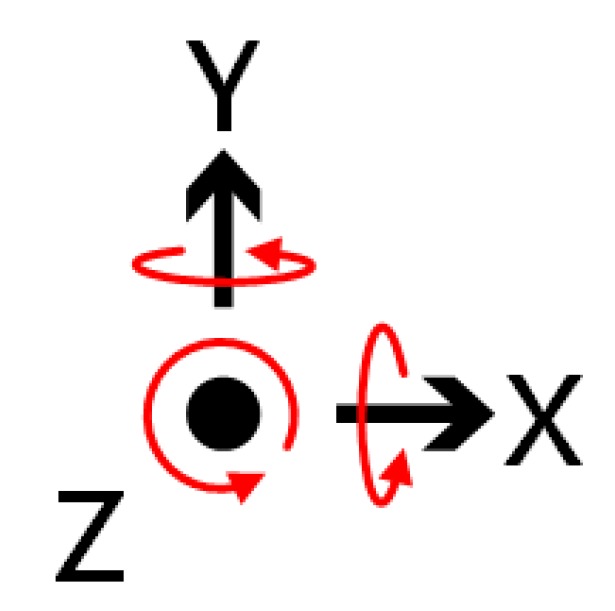
Gyro rotation direction diagram.

The gyroscope is mainly designed to measure the angular velocity, based on the theory of conservation of angular momentum. The angular velocity of rotation shown in Figure 15 is usually expressed in dps (°/s). Since the robot is traveling in a horizontal state, we mainly do angular offset compensation for the *Z*-axis. If only the *Z*-axis is rotated, then the *X* and *Y* axes output is 0 and the *Z*-axis output is the angular velocity of rotation.

We first calculate the angular velocity $v_{z}$ along *Z*-axis by

(17) $$v_{z}(t)=gz(t)/gyrosen$$

where *gz*(*t*) is the raw value read by the gyroscope and *gyrosen* is the sensitivity of the gyroscope. Then, the rotation angle $\phi_{gyro}$ along *z*-axis is calculated by

$$\phi_{gyro}=\int_{t}v_{z}(t)dt$$

There are advantages and disadvantages in both methods. Accelerometers are prone to error interference during fast vibration (high frequency). The gyroscope is prone to drift problems in an almost stationary (low frequency) state. In order to estimate collision offset, we introduce a parameter α(0<α<1) in an equation defined by

$$\phi =\alpha \phi_{acce}+(1-\alpha )\phi_{gyro}$$

**Figure 16:**
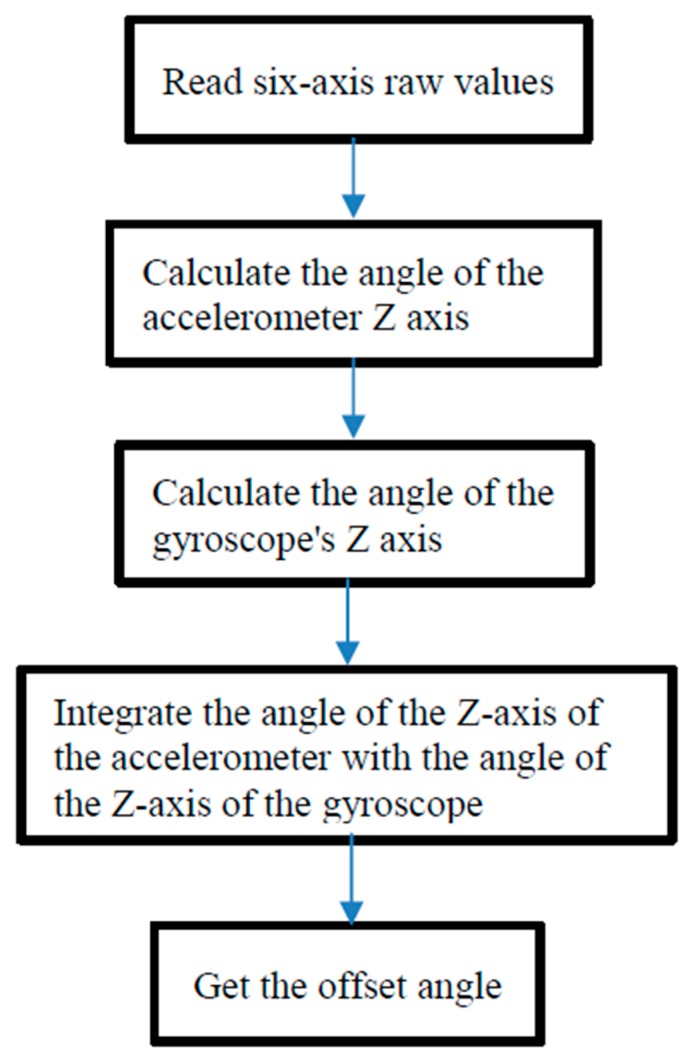
Operation flowchart of six-axis sensors.

Accordingly, the collision angle error compensation is completed. The detail of six-axis sensor usage process is shown in Figure 16.

### 3.5. Avoid Obstacles with Ultrasonic Sensors

**Figure 17:**
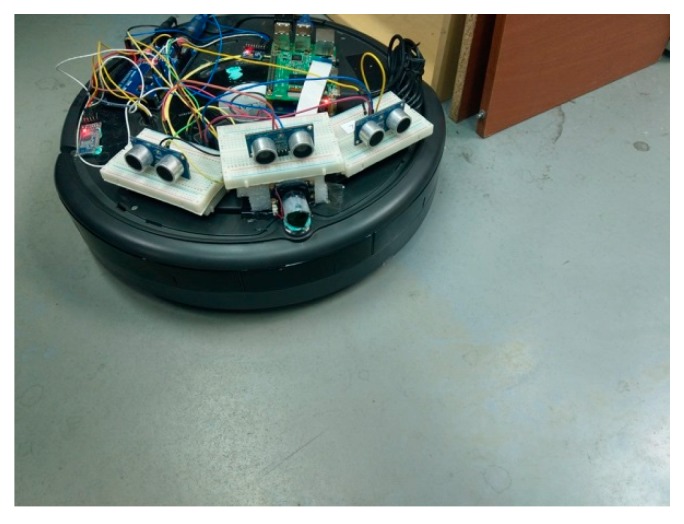
Three ultrasonic sensors installed on the clean robot.

**Figure 18:**
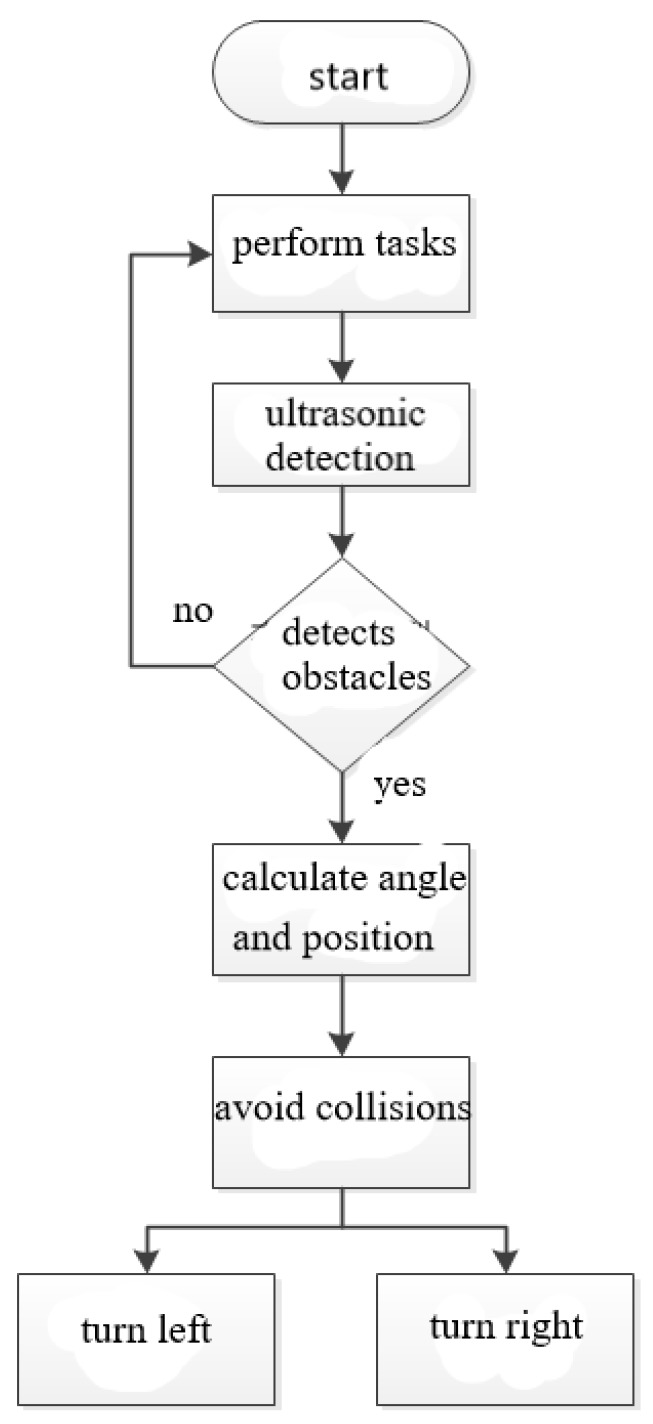
Operation flowchart of ultrasonic sensors.

When the speed of the clean robot is fast, the collision of the object will result in a large angular offset due to the impact force and the six-axis sensor detection speed cannot keep up with the compensation failure. To solve this problem, as shown in Figure 17 and Figure 18, we use the three ultrasonic sensors that are installed on the clean robot to immediately perform the direction change action when the cleaning robot detects obstacles around it, so as to avoid collisions with obstacles.

### 3.6. The proposed Machanism

**Figure 19:**
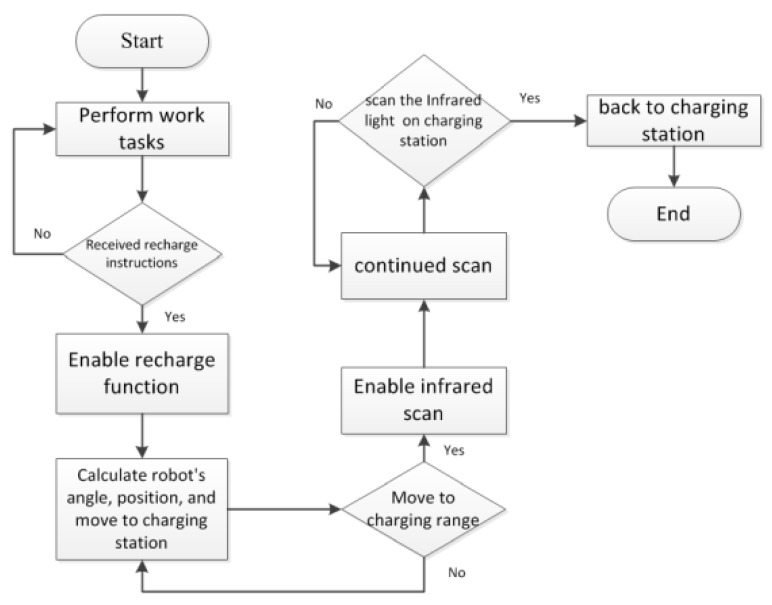
Auto-recharging mechanism for cleaning robot.

This paper mainly enables the robot automatically recharging with high efficiency. Based on subsections A-F, the entire automatic recharging mechanism is proposed, as shown in Figure 19, which is presented, as follows:

Step 1. Enable clean robot and perform certain tasks.
Step 2. Enable recharge instruction. If clean robot receives the recharging instruction, we determine the current position of clean robot and enable recharge function proposed in step 3 and step 4. Otherwise go back to step 1.
Step 3. Setting a threshold to enable six-axis sensor or ultrasonic. Six-axis sensor is enabled to compensate the angle error caused by collision when the travel speed is slow while ultrasonic is enabled to avoid obstacles when the travel speed is fast.
Step 4. Calculate clean robot’s angle and capture infrared spot via the HoughCircles function to judge whether the charging dock is currently on the left or the right side of the robot according to the current estimated angle and distance, and proceeds in the direction of the judgment. This action repeats until the infrared spot changes from one to two, indicating that it enters the infrared signal range (60° range). Once the infrared spot changes from one to two, go to step 5.
Step 5. Enable infrared scanning until the robot returns to charging correctly.

## 4. Experimental Results

**Figure 20:**
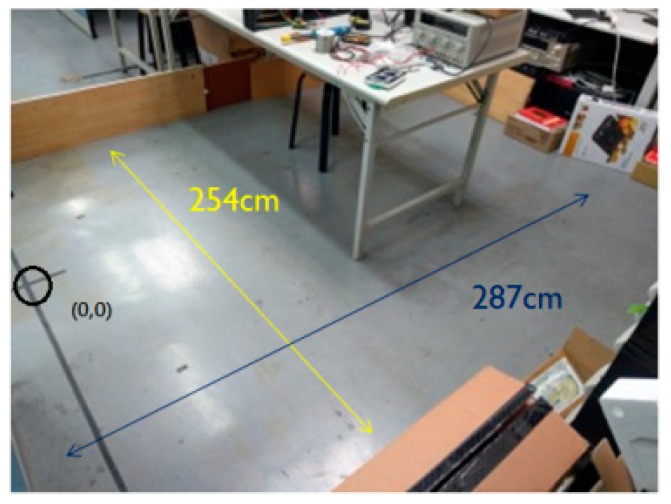
Experimental environment.

The experimental environment is 275 cm long and 254 cm wide with obstructions, as shown in Figure 20. The black circle is the starting position of the clean robot, and the charging dock placed behind the black circle.

### 4.1. Collision Angle Compensation

In order to verify that the angle compensation of the six-axis accelerometer has not been added, we compare the robot’s actual position and angle per minute. The experimental results are shown in Table 9. It can be found that the actual position, angle, and estimated position and angle are greatly different. In order to solve this problem, a six-axis accelerometer is added. The corrected results are shown in Table 10. After the addition of the six-axis sensor, the collision angle compensation is smaller than the original without added angle error, and the estimated position is within 50 cm from the actual position.

Although the accuracy of the traveling speed of 50 (mm/s) is improved after being compensated by the six-axis sensor, in comparison with the traveling speed of 200 (mm/s), as shown in Table 11, the error bias can be known. Big. The reason is that the faster the moving speed of the sweeping robot, the more the impact force will cause the angular offset to be large and the detection speed of the six-axis sensor cannot keep up, which will cause the compensation failure. To solve this problem, we use the three ultrasonic sensors. When the clean robot detects obstacles around it, then it can immediately perform the action of changing direction, avoiding collisions with obstacles, and avoiding angle errors that are caused by collisions.

Table 12 shows the results of the experiment after adding the ultrasonic sensor. It can be found that the problem of the angle caused by the collision after the addition of the ultrasonic sensor is greatly improved, but the longer the walking time, the position of the estimation is gradually caused by the problem of the error accumulation. There is a gap in the actual location.

### 4.2. Comparison between General-Type Auto-Recharging Mechanism and the Proposed Auto-Recharging Mechanism

This section compares the general-type recharge mechanism with the mechanism proposed in this paper. The general-type recharging mechanism has only the basic infrared detection recharging function without additional sensors and method for calculating its position. The mechanism that is proposed in this paper combines a variety of sensors to estimate the approximate position of the robot and thus proposed a new mechanism of automatic recharging. The comparison between the general-type recharging mechanism and the proposed mechanism is as follows.

**Figure 21:**
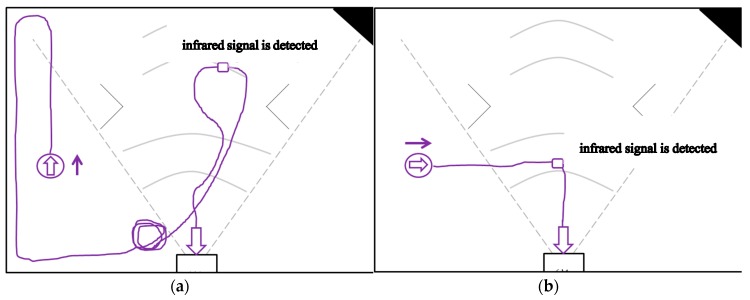

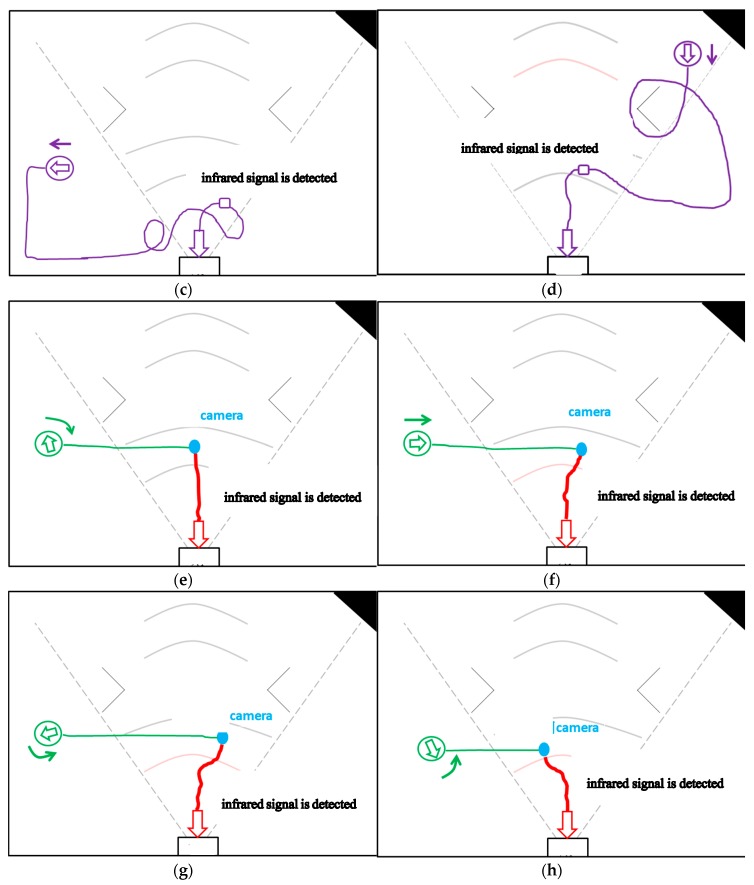
First part of experiment: the cleaning robot is placed outside the range of the infrared signal emitted by the infrared sensor of the docking station to back to charging by general-type and the proposed mechanisms. (**a**)Without detecting the infrared signal in the general-type mechanism, robot facing up bypasses until the infrared signal is detected. (**b**) Without detecting the infrared signal in the general-type mechanism, robot facing the right did not bypasses until the infrared signal is detected. (**c**) Without detecting the infrared signal in the general-type mechanism, robot facing the left bypasses until the infrared signal is detected. (**d**) Without detecting the infrared signal in the general-type mechanism, facing down bypasses until the infrared signal is detected. (**e**) By the proposed mechanism, robot facing up determines which direction should be followed and then finishes the recharge action. (**f**) By the proposed mechanism, robot facing the right determines which direction should be followed and then finishes the recharge action. (**g**) By the proposed mechanism, robot facing the left determines which direction should be followed and then finishes the recharge action. (**h**) By the proposed mechanism, robot facing down determines which direction should be followed and then finishes the recharge action.

The experiment is divided into two parts. In the first part, the cleaning robot is placed outside the range of the infrared signal emitted by the infrared sensor of the docking station to back to charging. That is, the cleaning robot is placed outside the range of the dotted fan in front of the docking station in Figure 21 to back to charging. As shown in Figure 21a–d, in the general-type mechanism, the circle with arrow inside is the beginning position of the cleaning robot, and the arrow is the direction of the current orientation. When the recharge instruction is enabled, the cleaning robot travels in the direction of the arrow. When the robot walks to the infrared signal range without detecting the infrared signal, it may bypasses until the infrared signal is detected. 

Figure 21e–h shows the automatic recharge mechanism that is proposed in this paper. The robot walks to the same position as the general-type mechanism, and then activates the recharging function. According to our proposed mechanism, it determines which direction should be followed and then finishes the recharge action. From these figures and Table 13, one can find that the efficiency of recharging is greatly improved. 

**Figure 22:**
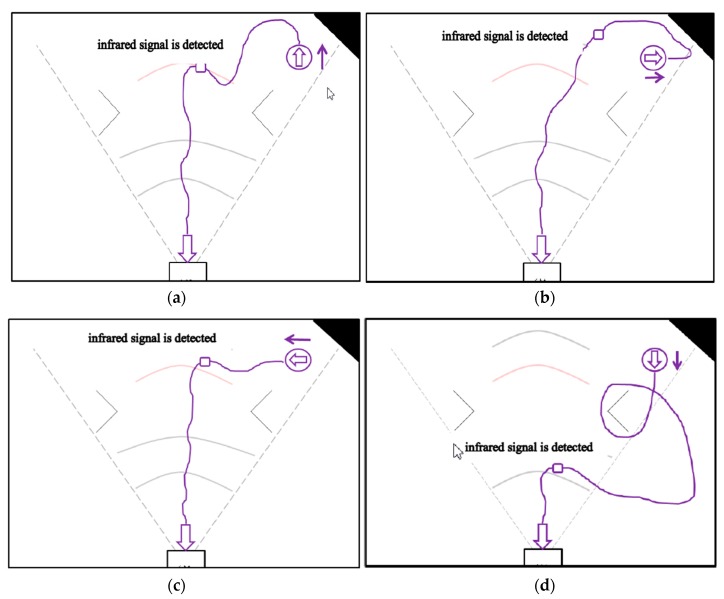

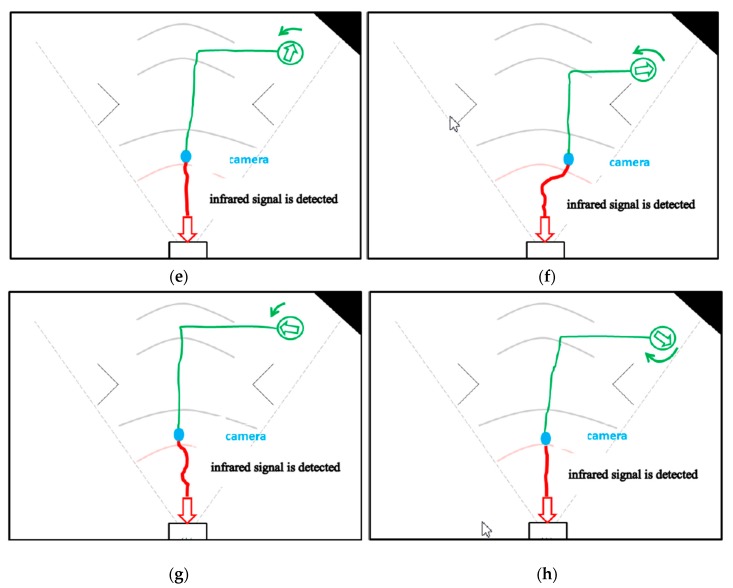
Second part of experiment: the cleaning robot is placed inside the range of the infrared signal emitted by the infrared sensor of the docking station to back to charging by general-type and the proposed mechanisms. (**a**) In the general-type mechanism, robot facing up bypasses until the infrared signal is detected. (**b**) In the general-type mechanism, robot facing the right bypasses until the infrared signal is detected. (**c**) In the general-type mechanism, robot facing the left bypasses until the infrared signal is detected. (**d**) In the general-type mechanism, robot facing down bypasses until the infrared signal is detected. (**e**) By the proposed mechanism, robot facing up determines which direction should be followed and then finishes the recharge action. (**f**) By the proposed mechanism, robot facing the right determines which direction should be followed and then finishes the recharge action. (**g**) By the proposed mechanism, robot facing the left determines which direction should be followed and then finishes the recharge action. (**h**) By the proposed mechanism, robot facing the right determines which direction should be followed and then finishes the recharge action.

The second part is the experiment performed inside the infrared signal range of the charging dock. Similarly, we compare the recharging path and time in different directions for the general-type mechanism and the proposed mechanism, as shown in Figure 22a–h and Table 14. It can be found that the automatic recharge time of the proposed mechanism is still less than the recharge time of the general mechanism.

## 5. Conclusions

This paper designs a high-efficiency automatic recharge mechanism to plan the method of automatic return charging through the travel distance, rotation angle, and so on. We made a variety of attempts on the way to find out the appropriate solution for robot travel distance, rotation angle, angle collision and other issues. Through the method that is proposed in this paper, the method of automatically returning the charging of the sweeping robot is planned, which is obviously more efficient than the method of returning charging to the general mechanism. 

In future research, we will continue to improve the accumulated error when walking for a long time even the machine learning method is used to compensate the error of the travel distance and the rotation angle of the sweeping robot. In addition, we will also improve the accuracy of image recognition.

## Figures and Tables

**Table 1:** Infrared signal range with and without adding three infrared LEDs.

	Without Adding Three Infrared LEDs	With Adding Three Infrared LEDs
**Infrared signal range (degree)**	60	60–90

**Table 2:** Specifications of MPU-6050 six-axis sensor.

Chip model	MPU-6050
Power supply	3–5 V
Communication protocol	I2C
Gyroscope range	±250, ±500,±1000,±2000°/s
Accelerometer range	±2, ±4, ±8, ±16g
16-bit AD converter/16-bit data output	

**Table 3:** Travel Distance—General Estimation.

Travel Time (s)	Estimated Travel Distance (cm)	Actual Travel Distance (cm)	Distance Error (cm)
10	5	6.45	1.45
20	10	12.8	2.8
30	15	18.2	3.2
40	20	24.1	4.1
50	25	30.2	5.2
60	30	36.2	6.2
70	35	42.1	7.1
80	40	47.8	7.8
90	45	53.7	8.7
100	50	59.4	9.4

**Table 4:** Comparison of travel distance after compensation.

Travel Time (s)	Estimated Travel Distance (cm)	Actual Travel Distance (cm)	Distance Error (cm)
10	6	5.9	−0.1
20	12	11.8	−0.2
30	18	18.2	0.2
40	23.97	24.2	0.23
50	29.95	30.1	0.15
60	35.93	36.3	0.37
70	41.92	42.4	0.48
80	47.9	48.6	0.7
90	53.88	54.3	0.42
100	59.86	60.4	0.54
120	71.63	71.4	−0.23
140	83.57	83.5	−0.07
160	95.51	96.2	0.69
200	119.39	118.5	−0.89
250	149.23	146	−3.23
300	179.08	175.8	−3.28
400	238.77	231.7	−7.07

**Table 5:** Rotation angle calculation—general estimation method.

Rotation Time (s)	Estimated Rotation Angle (°)	Actual Rotation Angle (°)	Angle Error (°)
1	42	29.5	12.5
1.5	63.825	44	19.825
2	85.1	58	27.1
2.5	106.375	72	34.375
3	127.65	86.5	41.15

**Table 6:** Comparison of rotation angle after compensation.

Rotation Time (s)	Estimated Rotation Angle (°)	Actual Rotation Angle (°)	Angle Error (°)
10	29.49	29	0.49
15	44.025	44	0.025
20	58.01	59	0.99
25	71.985	72	0.015
30	85.97	86.5	0.53
35	100.895	101.5	0.6
40	113.09	114	0.91
45	129.675	127.5	−2.175
50	144.07	142.3	−1.77
55	158.465	157.5	−0.965

**Table 7:** Distance table.

Distance between Robot and Docking Station (cm)	Radius (pixel)
10	25–33
20	25–27
30	25–27
40	19–23
50	18–20
60	18–20
70	17–19
80	15–18
90	14–17
100	12–15
110	9–12

**Table 8:** Collision angle offset without adding six-axis sensor.

Collision Number	Estimated Rotation Angle (°)	Actual Rotation Angle (°)	Angle Error (°)
1	58.4	57	1.4
2	350	352	2
3	281	286	5
4	212.8	218	5.2
5	161.4	170	8.6
6	110	120	10
7	41.3	52	8.7
8	332.8	347	14.2
9	264	277	13
10	195	206	11

**Table 9:** Comparison without Six-Axis Sensors.

Time (s)	Estimated Position	Actual Location	Distance Error (cm)	Estimated Angle (°)	Actual Angle (°)	Angle Error (°)
1	(−58.1, 157)	(−56.8, 164.3)	7.4	102.4	103	0.6
2	(94.3, 182)	(97.7, 208.2)	26.4	339.1	349	9.9
3	(55, 98.1)	(83.5, 106.6)	29.7	276	286	10
4	(−9.7, 44.5)	(17, 46)	26	118.6	134	15.4
5	(−23.1, 259.7)	(−53.6, 247.5)	32.8	329.9	357	27.1
6	(115.5, 105.6)	(131, 154.3)	51.1	241	276	34.3
7	(−101, 103.4)	(33.5, 58.2)	141.4	110.1	137	26.9
8	(−3.1, 263.4)	(−73.2, 236)	75.3	54.9	92	37.1
9	(−64.7, 196.9)	(−66.7, 142.2)	54.7	225.8	268	42.2
10	(−11.3, 11.6)	(95, 35.5)	108.7	351	33	−42
15	(−4.9, 50.2)	(137.2, 8.6)	148.1	248.1	253	4.9
20	(−68.3, 61.7)	(36.5, 53.4)	105.1	128.7	121	7.7
30	(−140.1, 14)	(135.6, 1.2)	275.7	109	96	13
40	(−148.2, 161.7)	(−83.2, 246.4)	106.8	19.6	8	11.6
50	(−137.4, 26.6)	(92.1, 3.4)	230.7	171.6	170	1.6

**Table 10:** Comparison of adding six-axis sensors (speed 50 mm/s).

Time	Estimated Position	Actual Location	Distance Error	Estimated Angle	Actual Angle	Angle Error
1	(−49, 146.1)	(−36, 152)	14.3	123.6	125	1.4
2	(−15.5, 192.3)	(−4.2, 194.4)	11.5	38.6	39	0.4
3	(111.8, 146.2)	(122, 133.8)	16.1	222	223	1
4	(−87.5, 58.1)	(−73.5, 56.6)	14.1	84.3	86	1.7
5	(−15.2, 252)	(−14.8, 243.5)	8.5	307	305	2
6	(137.2, 214.7)	(131.4, 200)	15.8	252.7	246	6.7
7	(22.5, 6.5)	(10, 9.5)	12.9	160.9	156	4.9
8	(34.2, 140.8)	(22.3, 139.8)	11.9	7.8	10	2.2
9	(77, 237.5)	(77.3, 230)	7.5	166.1	160	6.1
10	(−84.2, 165.3)	(−80.1, 170.2)	6.4	75	71	4
15	(125.4, 33.8)	(138.2, 35.4)	12.9	48.3	45	3.3
20	(−45.9, 102.4)	(−54.1, 98.6)	9	59.4	56	3.4
30	(154.6, 153.4)	(133.7, 148.6)	21.4	350.3	347	3.3
40	(−66.8, 79.2)	(−50.4, 95.3)	22.9	244.9	240	4.9
50	(118.8, 240.6)	(145.2, 227.3)	29.5	147.1	141	6.1

**Table 11:** Comparison of adding six-axis sensors (speed 200 mm/s).

Time	Estimated Position	Actual Location	Distance Error	Estimated Angle	Actual Angle	Angle Error
1	(−62.4, 155.1)	(−90.5, 223.7)	74.1	106.64	103	3.64
2	(32.3, 38)	(65.1, 72.4)	47.5	216.9	210	6.9
3	(79.1, 24.9)	(122.3, 78.3)	68.7	235.4	260	24.6
4	(−2.5, 204.2)	(−70.5, 244.9)	79.2	331.9	350	18.1
5	(86.2, 89.3)	(54.2, 85)	32.3	84.1	70	14.1
6	(11.7, 63.8)	(37.5, 5.6)	63.7	171.4	161	10.4
7	(26.7, 140.5)	(75.1, 168.6)	56	307.4	321	13.6
8	(−3.5, 236.5)	(−76, 243.2)	72.8	343.6	358	14.4
9	(−13.3, 102.7)	(−83.9, 31.1)	100.6	17	40	23
10	(124.3, 121.9)	(46, 141.5)	80.7	228.9	230	1.1
15	(−53.4, 108.4)	(−15.6, 133.2)	45.2	114.8	122	7.2
20	(33.9, 214.5)	(59.1, 175.3)	46.6	106.8	108	1.2
30	(148.6, 77.2)	(88.9, 114.9)	70.6	43.6	54	10.4
40	(−38.6, 87.5)	(−86.3, 115.7)	55.4	215.3	227	11.7
50	(146.3, 158.9)	(86.4, 133.8)	64.9	157.2	172	14.8

**Table 12:** Comparison of adding ultrasonic sensor (200 mm/s).

Time	Estimated Position	Actual Location	Distance Error	Estimated Angle	Actual Angle	Angle Error
1	(−103.5, 214.2)	(−101, 219)	5.4	193.3	192	1.3
2	(17.1, 236.7)	(14.6, 241.6)	5.5	179.9	177	2.9
3	(91.8, 165.8)	(95.8, 150.3)	16	112	111	1
4	(18.9, 155.3)	(14.3, 129.1)	26.6	112	110	2
5	(129.6, 120.1)	(123.5, 109)	12.7	96.3	96	0.3
6	(141.1, 128.7)	(134.1, 116.2)	14.3	268.1	268	0.1
7	(123.9, 113.8)	(110.3, 100.1)	19.3	114.3	114	0.3
8	(−87.6, 108.4)	(−99, 96.9)	16.6	97.6	94	3.6
9	(−54.9, 240.1)	(−64.3, 229)	14.5	354	351	3
10	(130.3, 97.9)	(123, 88.2)	11.9	98	96	2
15	(−103.3, 208.2)	(−80.5, 195.1)	26.3	282.2	277	5.2
20	(112.9, 125.3)	(133.1, 124.3)	20.2	75.5	73	2.5
30	(84.5, 30.3)	(53.7, 25.2)	31.2	347.9	341	6.9
40	(104.3, 107)	(118.4, 98)	16.7	128.6	127	1.6
50	(99.2, 160)	(120.6, 185.4)	33.2	91.8	90	1.8

**Table 13:** Time spent in the first part of experiment.

General-Type Mechanism	Recharging Time (s)	The Proposed Mechanism	Recharging Time (s)
(a)	76	(e)	36
(b)	20	(f)	26
(c)	35	(g)	33
(d)	112	(h)	29

**Table 14:** Time spent in the second part of experiment.

General-Type Mechanism	Recharging Time (s)	The Proposed Mechanism	Recharging Time (s)
(a)	32	(e)	31
(b)	43	(f)	30
(c)	29	(g)	30
(d)	41	(h)	35
